# First Results after Implantation of Hydrophilic and Hydrophobic Trifocal Intraocular Lenses: Visual and Optical Performance

**DOI:** 10.1155/2021/3514577

**Published:** 2021-12-18

**Authors:** Francisco Poyales, Ricardo Pérez, Israel López-Brea, Ying Zhou, Nuria Garzón

**Affiliations:** ^1^Miranza IOA Madrid, c/Galileo 104, Madrid, Spain; ^2^Optometry and Vision Department, Universidad Complutense de Madrid, c/Arcos de Jalón 118, Madrid, Spain

## Abstract

**Purpose:**

To compare postcataract surgery visual and optical performance between two trifocal intraocular lenses (IOLs) with the same optical design: a hydrophobic acrylic glistening-free IOL and a hydrophilic acrylic IOL.

**Methods:**

Patients were bilaterally implanted with either the hydrophobic or the hydrophilic IOL. The data of the patients' right eyes were evaluated. Visual quality assessments included refractive outcomes, monocular visual acuity (VA) at far, intermediate, and near distances, defocus curve, aberrations (spherical aberration (SA)), root mean square (RMS) of corneal, internal, and total higher-order aberrations (HOAs)), and tilt of IOL.

**Results:**

Fifty-one patients were included in the analysis: 26 patients implanted with the hydrophobic IOL and 25 patients implanted with the hydrophilic IOL. At 1 month, no statistically significant differences were found for monocular uncorrected and corrected VA at distance, distance-corrected VA at intermediate and near, defocus curve, manifest spherical equivalent, total SA, and RMS of the total, internal, and corneal HOA. The defocus curve of both groups showed a visual acuity of 0.3 logMAR or better in the intermediate range from 0.5 to −2.5 D of vergence level with no significant differences between the groups. Compared to the hydrophilic group, *y*-direction tilt was significantly higher in the hydrophobic group (*p*=0.027). The total tilt and *x*-axis tilt did not differ between the groups.

**Conclusion:**

Both IOLs demonstrated an excellent quality of vision and provided the patient with a wide range of vision.

## 1. Introduction

Monofocal intraocular lenses (IOLs) achieve excellent visual acuity (VA) results, but as the name suggests, only at one distance—usually far. As patients can no longer accommodate after IOL implantation, they need to use spectacles to see other distances in focus. The first-generation “bifocal” IOLs provided two focal points, one for far and the other for near vision, but thanks to an upsurge in computer, tablet, and smartphone use over recent years, there has been an upsurge in demand for IOLs that offer good intermediate vision too—something that bifocal IOLs cannot achieve [1]. In 2010, the first trifocal IOL (FineVision Micro F, PhysIOL, Liège, Belgium) was introduced [2], and numerous studies have shown that this IOL provides good far, intermediate, and near visual acuities (VA) and results in high levels of patient satisfaction [3–8].

The study reported here used two PhysIOL trifocal IOLs; both are based on the optical design of the FineVision Micro F IOL but differ in the material they are made from, which results in significant differences in the thickness of the IOLs. The POD F GF IOL is made from a hydrophobic acrylic glistening-free material, which should overcome the known disadvantages of conventional acrylic materials—both hydrophobic [9] and hydrophilic [10, 11].

This study compares the hydrophobic glistening-free POD F GF IOL with the hydrophilic POD F IOL in terms of optical quality after implantation in cataract patients.

## 2. Materials and Methods

This prospective, randomized, and controlled clinical study was performed to compare the quality of vision outcomes including monocular VA at far, intermediate, and near distances, refractive outcomes, defocus curve, aberrations, and IOL tilt in patients undergoing cataract surgery and bilateral implantation of either the FineVision POD F GF or FineVision POD F trifocal IOLs. The IOL model used for implantation was randomly chosen for each patient.

The clinical trial (NCT03347981) followed the provisions of the Declaration of Helsinki and was approved by the local ethics committee (CEIC Hospital Clínico San Carlos, Madrid, Spain).

### 2.1. IOL Models

Both IOL models used in this study are single-piece diffractive trifocal lenses, providing three focal points by combining two superimposed diffractive profiles, one with +1.75 D add power at the IOL plane for intermediate vision and another +3.50 D add power for near vision (the far focal point is created by nondiffracted light). The POD F GF and POD F IOLs both have an optic diameter of 6 mm and an overall diameter of 11.4 mm. Both IOLs have a 360° square edge around the optic in order to minimize posterior capsular opacification (PCO). The biconvex aspheric optic of the IOLs partly compensates for the positive corneal spherical aberrations (SAs). The main difference between the IOLs is the material they are manufactured with. The POD F GF IOL is made of glistening-free hydrophobic acrylate with a refractive index of 1.52. The POD F IOL is a 26% hydrophilic acrylic ultraviolet and blue light filtering lens with a refractive index of 1.46. Another minor difference between both IOL models is the design of the lens haptics. Both IOLs have double C-loop haptics with 5° angulation. The haptics of the POD F GF IOL have an additional wave-shaped structure that is intended to reduce the risk of adhesion between the haptics and the capsular bag during implantation.

### 2.2. Patients

A total of 51 patients were recruited for this study and were divided into two groups: the POD F GF group (*n* = 26) bilaterally implanted with the POD F GF IOL and the POD F group (*n* = 25) with the POD F IOL. A calculation of the sample size showed that this number of subjects per group was adequate to compare the optical quality between groups.

The focus of this paper is the presentation of the monocular visual quality of the patients. To avoid bias, the 1-month outcomes of patients' right eyes (OD) are presented here.

Cataractous patients with an age of 50 years or older were included after uneventful cataract surgery if they had no comorbidities, the desire for spectacle independence after surgery, realistic expectations, availability, willingness, and sufficient cognitive awareness to comply with examination procedures. Exclusion criteria were irregular astigmatism, regular astigmatism >1.0 D measured by automated keratometry or biometry or >1.25 D if the steep axis of the cylinder was between 90° and 120° in one or both eyes, acute or chronic disease or illness that would increase risk or confound study results, history of ocular trauma or prior ocular surgery including refractive procedures, capsule or zonular abnormalities that may affect postoperative centration or tilt of the IOL, pupil abnormalities, and AMD suspicious eyes.

### 2.3. Surgery

Cataract surgery was performed by one experienced surgeon (FP) using standard phacoemulsification and a 2.2 mm incision. All but four patients received a CTR in both eyes to increase the placement stability of the IOL and to avoid postoperative myopization. The Accujet 2.1 injector (Medicel, Thal, Switzerland) was used for all implantations to standardize surgically induced astigmatism.

### 2.4. Methods of Evaluation

Preoperative assessment included manifest refraction, corrected distance VA (CDVA), intraocular pressure, and corneal keratometry and biometry (IOLMaster 700, Carl Zeiss Meditec AG, Germany).

The main outcome measures at 1 month postoperatively included manifest refraction, prediction error, monocular uncorrected and corrected VA at far (UDVA and CDVA), distance-corrected intermediate VA (DCIVA), distance-corrected near VA (DCNVA), monocular defocus curve, and aberrometry.

Visual acuities at distance, intermediate, and near were measured at 4 m, 70 cm, and 35 cm, respectively, and were performed using Early Treatment Diabetic Retinopathy Study (ETDRS) charts (Precision Vision, USA). Distance-corrected visual acuities (CDVA, DCIVA, and DCNVA) were measured using subjective refraction for far distance.

Monocular defocus curves were generated by adding a defocus lens to the best-corrected refraction from +2.0 D to −4.0 D in steps of 0.5 D. With each defocus lens, VA was tested at 4 m using ETDRS charts.

The OPD-Scan III (Nidek Inc., Japan) was used to measure photopic and mesopic pupil sizes and aberrometry measurements including spherical aberrations (SA), root mean square (RMS) of the total, corneal, and internal higher-order aberrations (HOAs), and tilt.

### 2.5. Statistical Analysis

Data analysis was performed using Microsoft Excel (version 16.0) and the WinSTAT (version 2012.1.0.96) plug-in. Descriptive statistics were expressed as mean (±standard deviation (SD), median, and range). The Mann–Whitney *U* test was used to assess the significance of differences between groups. The Wilcoxon rank-sum test for paired data was performed to assess the significance of differences between examinations. A *p* value of less than 0.05 was considered statistically significant. The refractive prediction error was defined as the difference in achieved postoperative manifest SE and predicted SE.

Sample size was determined using the sealed envelope power calculator. To show no difference between the two study cohorts and with a drop out of 15%, the sample size calculation results in 25 patients per study group.

## 3. Results

Fifty-one right eyes were included in the study analysis: 26 eyes in the POD F GF group and 25 eyes in the POD F group. All patients had uneventful cataract surgery with IOL implantation and completed 1-month of follow-up. The demographics and IOL power are summarized in Table 1. There were no significant differences in patients' age, gender, or IOL power between the study groups (*p* > 0.05).

**Figure 1:**
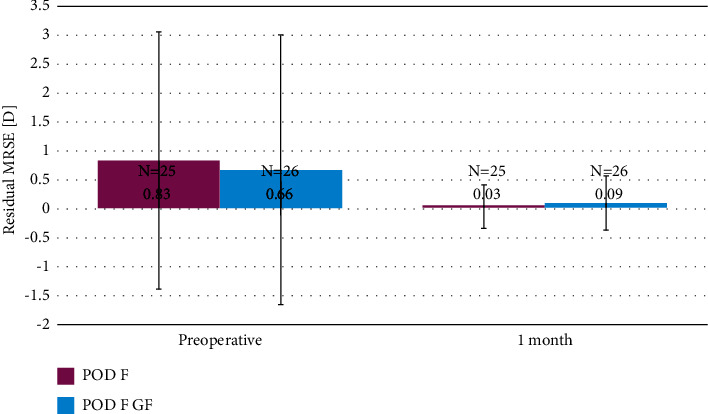
Mean refractive spherical equivalent preoperatively and 1 month (D).

As shown in Figure 1, the manifest refraction spherical equivalent (MRSE) at 1 month postoperatively did not differ statistically significantly between the groups (*p* = 0.299). Mean MRSE was 0.09 ± 0.47 D in the POD F GF group and 0.03 ± 0.38 D in the POD F group after 1 month; compared with preoperative values (POD F GF: 0.66 ± 2.35 D; POD F: 0.83 ± 2.24 D), there was a numerical improvement, but not a statistically significant one (*p*=0.112 in the POD F GF group and *p*=0.058 in the POD F group).

**Figure 2:**
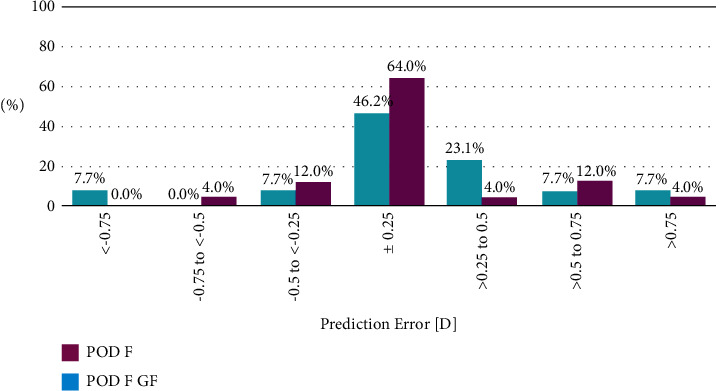
Distribution of prediction error 1 month postoperatively (% of eyes).

At 1 month postoperatively, both study groups turned out slightly hyperopic (0.11 ± 0.44 D in the POD F GF group and 0.07 ± 0.38 D in the POD F group) with no significant differences between the groups (*p*=0.418).  Figure 2 shows the distribution of prediction error: 86% of eyes in the POD F GF group and 80% of eyes in the POD F group were within ±0.5 D of the targeted MRSE.

Visual acuities at 1 month postoperatively are shown in Table 2. No significant differences were observed between the POD F GF and POD F groups.

**Figure 3:**
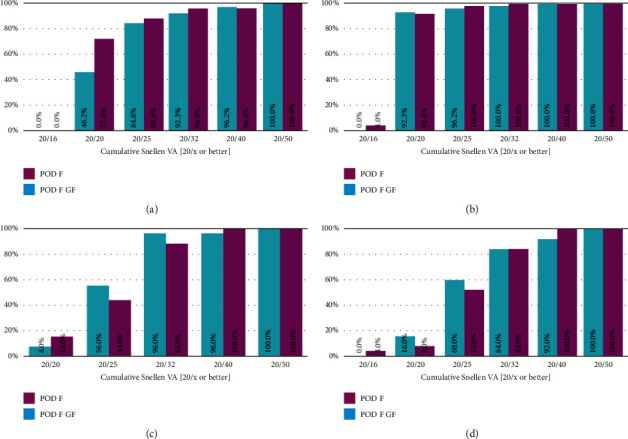
Cumulative distribution of uncorrected distance visual acuity (UDVA) (a), corrected distance visual acuity (CDVA) (b), distance-corrected intermediate visual acuity at 70 cm (DCIVA) (c), and distance-corrected near visual acuity at 35 cm (DCNVA) (d). Monocular visual acuities are shown 1 month postoperatively for eyes implanted with a POD F GF IOL or a POD F IOL.

Figure 3 shows the cumulative distribution of UDVA, CDVA, DCIVA, and DCNVA for both groups.

Almost 85% of eyes in the POD F GF group and 88% of eyes in the POD F group had a UDVA of 20/25 or better, and 100% of eyes in both groups had a UDVA of 20/50. CDVA was 20/25 or better in about 96% of eyes in the POD F GF group and in all eyes (100%) in the POD F group. DCIVA and DCNVA were better than 20/40 in 96% and 92% of eyes in the POD F GF group and all eyes (100%) of the POD F group, respectively.

**Figure 4:**
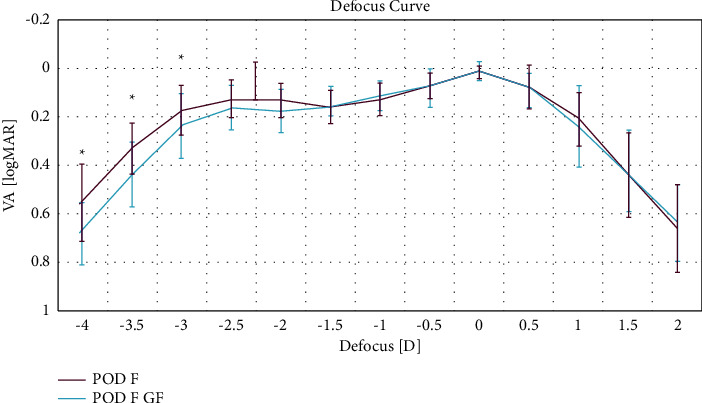
Monocular defocus curves 1 month postoperatively for eyes implanted with a POD F GF IOL or a POD F IOL.

The mean monocular defocus curves with the standard deviations are shown in Figure 4.

Maximal VA values were obtained in both groups at a vergence level of 0.0 D, corresponding to the distance vision. Between distance and near visions (i.e., defocus levels between 0.5 and −2.5 D), all eyes of both groups displayed a VA of 0.3 logMAR or better. Eyes that received the POD F IOL had significantly better VA at the vergence levels −4.0 D, −3.5 D, and −3.0 D (*p*=0.013, *p*=0.014, and *p*=0.042) compared to eyes in the POD F GF group. For all other vergence levels, there were no significant differences between both groups (*p* > 0.05).

The photopic and mesopic pupil sizes did not significantly differ between the groups (*p*=0.510 and *p*=0.279, respectively). The SA, RMS of the total, internal, and corneal HOA, and x-axis, *y*-axis, and total tilt of the lens, as measured by the OPD-Scan III, are shown in Table 3. The tilt in *y*-direction was significantly higher in the POD F GF group than in the POD F group (*p*=0.027). Spherical aberrations, HOAs, *x*-axis tilt, and total tilt did not differ between the groups.

In the course of the study, three adverse events (AEs) and one serious adverse event (SAE) occurred within 1 month. All of them were classified as “recovered” after study completion.

In the course of the detailed slit-lamp examination after 1 month, one eye of the POD F GF group showed “mild” PCO (i.e., no need for Nd : YAG treatment). In three eyes, dry eye was diagnosed (two of the eyes were in the POD F group and the remaining one in the POD F GF group). Two eyes (both from the POD F group) developed uveitis (anterior uveitis and slight uveitis, respectively) 1 month after surgery. No anterior capsular fibrosis was detected in any case.

## 4. Discussion

The present clinical trial demonstrated that eyes implanted with either POD F GF IOL or the POD F IOL achieved a very good postoperative quality of vision. Both IOLs provided similarly good monocular UDVA outcomes, with 85% of the POD F GF group and 88% of the POD F group achieving VA of 20/25 or better. A DCIVA of 20/40 or better was achieved in 96% of the POD F GF group and 100% of the POD F group. The DCNVA was 20/40 or better in 92% of the POD F GF group and 100% of the POD F group. The distance VA results for both the POD F GF IOL and POD F IOL achieved in this study are comparable to those of other studies that examined trifocal IOL models [5, 6, 12–16]. Regarding intermediate and near VAs, the results observed with trifocal IOLs in the literature vary and range from 0.1 logMAR or better for DCIVA and DCNVA [6, 12, 15, 17] to visual acuities considerably below 0.1 logMAR [5, 17, 18]. The DCIVA and DCNVA of our study are within the given ranges of the literature data. However, direct comparisons are difficult due to different measurement methods (VA charts and distances) and different sizes of the study populations. The performance at intermediate and near distances in this study is slightly below that of other studies with the same or similar IOLs. Studies with the same or similar IOL models have shown that intermediate visual acuity improved between 1 and 3 months, and near visual acuity increased until 6 months [12].

There were no significant differences in the visual results of the eyes that received the POD F GF and POD F IOLs in this study. So we are confident in concluding that the different materials do not affect the optical performance of the IOLs. No glistenings were observed in either the POD F GF group or the POD F group during the 1-month follow-up period, although this is a short follow-up period, and much longer-term follow-up would be required for the long-term behavior of this material.

The defocus curves in the POD F GF and POD F groups showed two peaks: one at the vergence level 0.0 D (corresponding to distance vision) and one at −2.5 D (corresponding to 40 cm). There was no considerable decrease in intermediate VA, in the defocus curve range between −2.5 D and 0.0 D, reflecting the clear advantage trifocal IOLs have over bifocal IOLs, which are associated with a V-shaped defocus curve, decreasing considerably in the intermediate range between about −0.5 D and −1.5 D [17, 19–21].

In the intermediate range, both groups achieved VA values between 0.1 and 0.17 logMAR. For the POD F group, significantly better VA at near distance between −4.0 D and −3.0 D of defocus was observed relative to the POD F GF group, although there is the possibility that this can be attributed to the (numerically, but not significantly) slightly smaller pupil sizes in the POD F group which may have led to improved performance at close range [21].

The mean total HOA RMS is an important parameter to measure when assessing the optical performance of an IOL; this was also comparable between the groups: 0.29 ± 0.13 *µ*m in the POD F GF group and 0.26 ± 0.12 *μ*m in the POD F group. Whether or not HOAs need to be corrected in cataract surgery is a challenge for which no final answer has yet been found, since the required accuracy of centration of these wavefront-corrected IOLs is an obvious concern. In most cases, they degrade the quality of vision. Otherwise, they may have a beneficial effect. In the case of presbyopia, certain amounts of HOAs (above all SAs) have the potential to increase the depth of field without having a negative impact on visual acuity. If the natural lens is removed during cataract surgery and replaced by an artificial lens, the internal aberrations and accordingly the aberrations of the entire optical system change. Both IOL models used in this study partly compensate for the positive SA of the cornea by providing a SA of −0.11 *μ*m at a 5 mm pupil. Thus, the IOLs are close to being “aberration-free” IOLs, which also means less sensitivity to decentration and tilt, but does not necessarily lead to any disadvantage in imaging quality compared to the extended depth of focus or bifocal IOLs [22]. No statistically significant differences in SA between the study groups were found.

This study focused on the assessment of monocular visual performance after the implantation of trifocal IOLs. Binocular evaluation was omitted to avoid bias but will be part of a future analysis in connection with a longer follow-up.

In the current study, the POD F and the POD F GF IOLs both demonstrated very good visual results and good refractive predictability, with only small deviations towards hyperopia over a 1-month follow-up period. During this short follow-up period, no disadvantages associated with the hydrophobic GF biomaterial were observed in the POD F GF IOL, compared with the hydrophilic material-containing POD F IOL. All surgeries were uneventful, which is an indication of easy handling and implantation of both IOL models.

## 5. Conclusion

Based on the clinical and safety data shown in this study, it can be concluded that the implantation of both trifocal lenses within their intended use seems to be a safe and effective option to compensate for presbyopia in the course of cataract surgery. However, future long-term postmarket clinical follow-up studies and postmarket surveillance activities are necessary to confirm these outcomes and to evaluate postoperative spectacle independence and patient satisfaction.

## Figures and Tables

**Table 1:** Patient demographics and IOL power per group.

	POD F GF	POD F	*p* value^Ɨ^
*Gender (n)*			
Female	23	21	0.703
Male	3	4	

*Age (years)*			
Mean ± SD	66.0 ± 6.9	65.0 ± 6.3	0.553
Median	67.5	66.0	
Min/max	52.7/83.7	47.1/75.2	

*IOL power (D)*			
Mean ± SD	22.6 ± 2.0	21.8 ± 2.3	0.094
Median	23.0	22.0	
Min/max	17.0/25.5	16.0/27.5	

*Target MRSE (D)*			
Mean ± SD	0.00 ± 0.10	0.00 ± 0.20	0.992
Median	0.0	0.0	
Min/max	−0.20/0.20	−0.60/0.20	

^Ɨ^POD G GF IOL vs. POD F IOL. SD = standard deviation; min = minimum; max = maximum; IOL = intraocular lens; MRSE = mean refraction spherical equivalent.

**Table 2:** Monocular postoperative visual acuities (logMAR) at 1 month per group.

	POD F GF	POD F	*p* value^Ɨ^
*UDVA*			
Mean ± SD	0.08 ± 0.10	0.05 ± 0.10	0.097
Median	0.10	0.00	
Min/max	0.40/0.00	0.40/0.00	

*CDVA*			
Mean ± SD	0.01 ± 0.04	0.00 ± 0.04	0.661
Median	0.00	0.00	
Min/max	0.20/0.00	0.10/-0.10	

*DCIVA*			
Mean ± SD	0.12 ± 0.08	0.12 ± 0.09	0.887
Median	0.10	0.10	
Min/max	0.40/0.00	0.30/0.00	

*DCNVA*			
Mean ± SD	0.12 ± 0.12	0.12 ± 0.10	0.877
Median	0.10	0.10	
Min/max	0.40/0.00	0.30/-0.10	

^Ɨ^POD G GF IOL vs. POD F IOL. UDVA = uncorrected distance visual acuity; CDVA = corrected distance visual acuity; DCIVA = distance-corrected intermediate visual acuity; DCNVA = distance-corrected near visual acuity; SD = standard deviation; min = minimum; max = maximum.

**Table 3:** Corneal spherical aberration, higher-order aberrations, and IOL tilt at 1 month per group.

	POD F GF	POD F	*p* value^Ɨ^
*SA (μm)*			
Mean ± SD	0.23 ± 0.19	0.27 ± 0.14	0.451
Median	0.25	0.27	
Min/max	−0.08/0.57	−0.10/0.56	

*RMS of total HOA (μm)*			
Mean ± SD	0.29 ± 0.13	0.26 ± 0.12	0.300
Median	0.30	0.25	
Min/max	0.08/0.66	0.09/0.54	

*RMS of corneal HOA (μm)*			
Mean ± SD	0.35 ± 0.31	0.27 ± 0.16	0.451
Median	0.27	0.24	
Min/max	0.09/1.48	0.10/0.87	

*RMS of internal HOA (μm)*			
Mean ± SD	0.36 ± 0.33	0.27 ± 0.15	0.155
Median	0.29	0.21	
Min/max	0.10/1.56	0.14/0.73	

*x-axis tilt (μm)*			
Mean ± SD	−0.11 ± 0.29	−0.15 ± 0.29	0.806
Median	−0.12	−0.15	
Min/max	−0.54/0.91	−0.52/0.13	

*y-axis tilt (μm)*			
Mean ± SD	0.10 ± 0.20	−0.01 ± 0.14	0.027^*∗*^
Median	0.09	−0.01	
Min/max	−0.35/0.67	−0.34/0.20	

*Total tilt (μm)*			
Mean ± SD	0.31 ± 0.21	0.24 ± 0.13	0.337
Median	0.25	0.22	
Min/max	0.05/0.98	0.08/0.56	

^Ɨ^POD G GF IOL vs. POD F IOL. ^*∗*^Significant at level *α* < 0.05. SA = spherical aberrations; HOA = higher-order aberrations; RMS = root mean square; SD = standard deviation; min = minimum; max = maximum.

## Data Availability

The authors do not intend to share individual patient raw data for reasons of data protection.

## References

[1] Jin S., Friedman D. S., Cao K. (2019). Comparison of postoperative visual performance between bifocal and trifocal intraocular Lens based on randomized controlled trails: a meta-analysis. *BMC Ophthalmology*.

[2] Gatinel D., Pagnoulle C., Houbrechts Y., Gobin L. (2011). Design and qualification of a diffractive trifocal optical profile for intraocular lenses. *Journal of Cataract & Refractive Surgery*.

[3] Oliveira R. F., Vargas V., Plaza-Puche A. B., Alió J. L. (2020). Long-term results of a diffractive trifocal intraocular lens: visual, aberrometric and patient satisfaction results. *European Journal of Ophthalmology*.

[4] Singh B., Sharma S., Dadia S., Bharti N., Bharti S. (2019). Comparative evaluation of visual outcomes after bilateral implantation of a diffractive trifocal intraocular lens and an extended depth of focus intraocular lens. *Eye and Contact Lens: Science and Clinical Practice*.

[5] Poyales F., Garzón N., Pizarro D., Cobreces S., Hernández A. (2019). Stability and visual outcomes yielded by three intraocular trifocal lenses with same optical zone design but differing material or toricity. *European Journal of Ophthalmology*.

[6] Ferreira T. B., Ribeiro F. J. (2019). Prospective comparison of clinical performance and subjective outcomes between two diffractive trifocal intraocular lenses in bilateral cataract surgery. *Journal of Refractive Surgery*.

[7] Ferreira-Ríos I., Zuñiga-Posselt K., Serna-Ojeda J. C., Chávez-Mondragón E. (2018). Objective and subjective results following implantation of the FineVision trifocal intraocular lens in Mexican patients. *International Ophthalmology*.

[8] de Carneros-Llorente A. M., de Carneros A. M., de Carneros-Llorente P. M., Jiménez-Alfaro I. (2019). Comparison of visual quality and subjective outcomes among 3 trifocal intraocular lenses and 1 bifocal intraocular lens. *Journal of Cataract & Refractive Surgery*.

[9] Gregori N. Z., Spencer T. S., Mamalis N., Olson R. J. (2002). In vitro comparison of glistening formation among hydrophobic acrylic intraocular lenses. *Journal of Cataract & Refractive Surgery*.

[10] Chang A., Kugelberg M. (2017). Posterior capsule opacification 9 years after phacoemulsification with a hydrophobic and a hydrophilic intraocular lens. *European Journal of Ophthalmology*.

[11] Li Y., Wang J., Chen Z., Tang X. (2013). Effect of hydrophobic acrylic versus hydrophilic acrylic intraocular lens on posterior capsule opacification: meta-analysis. *PLoS One*.

[12] Nagy Z. Z., Popper-Sachetti A., Kiss H. J. (2019). Comparison of visual and refractive outcomes between hydrophilic and hydrophobic trifocal intraocular lenses sharing the same optical design. *Journal of Cataract & Refractive Surgery*.

[13] Cochener B., Vryghem J., Rozot P. (2014). Clinical outcomes with a trifocal intraocular lens: a multicenter study. *Journal of Refractive Surgery*.

[14] Cochener B., Vryghem J., Rozot P. (2012). Visual and refractive outcomes after implantation of a fully diffractive trifocal lens. *Clinical Ophthalmology*.

[15] Vryghem J. C., Heireman S. (2013). Visual performance after the implantation of a new trifocal intraocular lens. *Clinical Ophthalmology*.

[16] Bilbao-Calabuig R., Llovet-Rausell A., Ortega-Usobiaga J. (2017). Visual outcomes following bilateral lmplantation of two diffractive trifocal intraocular lenses in 10 084 eyes. *American Journal of Ophthalmology*.

[17] Mojzis P., Kukuckova L., Majerova K., Ziak P., Piñero D. P. (2017). Postoperative visual performance with a bifocal and trifocal diffractive intraocular lens during a 1-year follow-up. *International Journal of Ophthalmology*.

[18] Alió J. L., Montalbán R., Peña-García P., Soria F. A., Vega-Estrada A. (2013). Visual outcomes of a trifocal aspheric diffractive intraocular lens with microincision cataract surgery. *Journal of Refractive Surgery*.

[19] Shen Z., Lin Y., Zhu Y., Liu X., Yan J., Yao K. (2017). Clinical comparison of patient outcomes following implantation of trifocal or bifocal intraocular lenses: a systematic review and meta-analysis. *Scientific Reports*.

[20] Liu X., Xie L., Huang Y. (2018). Comparison of the visual performance after implantation of bifocal and trifocal intraocular lenses having an identical platform. *Journal of Refractive Surgery*.

[21] Kondylis G., Klavdianou O., Palioura S. (2019). Multifocal and extended depth of focus intraocular lenses. *Annals of Eye Science*.

[22] Gatinel D., Loicq J. (2016). Clinically relevant optical properties of bifocal, trifocal, and extended depth of focus intraocular lenses. *Journal of Refractive Surgery*.

